# Effect of dosage of orally administered 17α-methyltestosterone on sex reversion of the yellowtail tetra *Astyanax lacustris* (Lütken, 1875)

**DOI:** 10.1590/1984-3143-AR2022-0080

**Published:** 2023-03-06

**Authors:** Renata da Silva Farias, Karolayne Ribeiro da Silva Oliveira, Marília Espíndola de Souza, Dijaci Araújo Ferreira, Alluanan Adelson do Nascimento Silva, Valdemiro Amaro da Silva, Rex Dunham, Maria Raquel Moura Coimbra

**Affiliations:** 1 Departamento de Pesca e Aquicultura, Universidade Federal Rural de Pernambuco, Recife, PE, Brasil; 2 Colégio Agrícola Dom Agostinho Ikas, Universidade Federal Rural de Pernambuco, São Lourenço da Mata, PE, Brasil; 3 Departamento de Medicina Veterinária, Universidade Federal Rural de Pernambuco, Recife, PE, Brasil; 4 School of Fisheries, Aquaculture and Aquatic Sciences, Auburn University, Auburn, AL, USA

**Keywords:** biotechnology, neomale, phenotypic male, sex reversal

## Abstract

The females of yellowtail tetra (*Astyanax lacustris*), known as the freshwater sardine, are approximately 1.33 times larger than males, and thus, all-female monosex culture would increase production and reduce size variability. The present work aimed to identify the optimal dose of 17α-methyltestosterone (MT) to be used in the masculinization of *A. lacustris* for indirect sex reversal. Three different concentrations of MT (20, 40, and 60 mg/kg of feed in the diet) were fed to the fry for 30 days. Thirty adult individuals from each treatment, including the control (0 mg MT/kg), were evaluated for gonadal development, morphological and histological sexual identification, zootechnical performance, and the possible genotoxic effect caused by prolonged exposure to MT. MT significantly (*P*<0.01) affected the differentiation of the gonads, with the presence of possible inhibitory effects in all treatments. Intersex individuals were present in the 20 and 60 mg MT/kg treatments. All treatments were able to masculinize *A. lacustris* and the treatment with the lowest hormone concentration produced the highest percentage of males 76.7%, while the control had 46.7% males. The presence of erythrocyte nuclear alterations indicated a possible cytotoxic effect of MT in treatments 40 and 60 mg MT/kg, however, the use of the hormone did not affect the growth and the survival of the individuals. Thus, the use of MT is a viable option for obtaining neomales as a first step into the production of all-female progenies.

## Introduction

The yellowtail tetra, *Astyanax lacustris*, popularly known in Brazil as freshwater sardine, piaba or lambari, belongs to the order Characiforme, the Characidae family, and the Tetragonopterinae subfamily (Barbieri et al., 1982). They are small fish, benthopelagic, omnivorous with an insectivorous tendency and a short life cycle (Agostinho et al., 2004; Silva et al., 2012).

The yellowtail tetra aquaculture is a recent activity, and its production technology is still under development. It is an alternative financial source for small rural producers in Brazil (Fonseca et al., 2017), reaching 631 tons in 2020 (IBGE, 2020). It has a great potential to be developed in a sustainable way, promoting socio-economic development, improving food security, and developing local communities in Brazil. The yellowtail tetra has its own market niche. It has been appreciated as appetizer in restaurants, has been used as live bait for sport fishing and it is a feasible alternative for live bait in commercial tuna fishing (Valladão et al., 2018; Valenti et al., 2021).

Yellowtail tetra reaches sexual maturity in captivity in a period of up to four months, allowing several spawnings throughout the year (Garutti, 2003; Porto-Foresti et al., 2005; Abimorad and Castellani, 2011). Adult individuals show evident sexual dimorphism during the spawning season, males present an elongated body and are smaller than females, the latter also presenting a bulging belly (Súarez et al., 2017; Stevanato and Ostrensky, 2018).

*Astyanax lacustris* is a gonochoristic specie, i.e., each individual organism is either male or female (Leonard, 2019). Sex differentiation moment and sex determination system are not well known/described for *A. lacustris*, but in a sister species, *A. altiparanae*, it occurs at 58 days post-hatching (dph) and 73 dph for ovaries and testis, respectively (Adolfi et al., 2015), and it presents a XX/XY sex determination system (Lázaro et al., 2021).

The heterogeneity in the harvest size of adult males and females is a major problem faced by farmers. In order to decrease this variability and increase the efficiency and profitability of the cultivation of this species, the use of all-female progeny resulted in a higher growth rate when compared to traditional culture (Navarro et al., 2006; Bem et al., 2012). All-female progeny can be obtained via hormone sex reversal technique in a direct or indirect way, administering hormones by immersion or in the diet during the period of histological sexual differentiation. For the direct hormonal sex reversal, the estrogen hormone (estradiol-17β) is supplied during the early stages of development. Yet for the indirect form, genotypic females are masculinized with the androgen hormone (17α-methyltestosterone) becoming neomales, capable of producing sperm to fertilize oocytes of normal females (phenotypic and genotypic females) (Toledo-Filho et al., 1996; Dunham, 2011; Weiss et al., 2018a). In theory, the progeny resulting from the crossing of neomales (XX) with natural females (XX) will be composed exclusively of females.

Despite being widely used, the direct sexual reversion technique during larviculture is criticized for the potential risk of environmental contamination due to the periodic use of hormones (Johnstone et al., 1983; Pandian and Sheela, 1995; Thanasupsin et al., 2021) and the fact that fish for human consumption have received hormones in some stage of their life. Sex reversal and breeding, on the other hand, may be less harmful to the environment, as the frequency of hormone used will be lower, since it will only be used for the formation of the broodstock and not to reverse the offspring every new cycle, as in the direct sex reversion (Toledo-Filho et al., 1996; Piferrer, 2001; Dunham, 2011). In addition, the use of hormones for sex reversal can cause negative effects on growth, survival, and gonadal development, if a threshold dose is exceeded (Shen et al., 2015; Fatima et al., 2016). Thus, the timing, duration, and dosage of the hormone used to induce sex reversal are species-specific, and the effects and efficacy of its use for indirect sex reversal in yellowtail tetra, *A. lacustris*, have yet to be elucidated.

Studies using MT in the feed to produce neomales, which later can be used to produce an all-female population, have been done in different fish species, such as the Neotropical species jundiá (*Rhamdia quelen*) obtaining 72% of males at a dose of 60 mg/kg (Weiss et al., 2018a), and for rainbow trout (*Oncorhynchus mykiss*) 96% of males were achieved at 2 mg/kg MT (Kashe et al., 2021). Also, for silver perch (*Bidyanus bidyanus*) 100% of males were obtained using 9 mg/kg (Sulaeman and Fotedar, 2017).

Thus, the aim of this work was to evaluate the effect of adding different concentrations of 17α-methyltestosterone in the diet of yellowtail tetra *A. lacustris*, in order to establish a masculinization protocol for obtaining XX neomales.

## Methods

### Sexual reversal

To determine the optimal dose of the masculinizing hormone 17α-methyltestosterone (MT), three different concentrations of the hormone in the diet were tested. The experiment was conducted at the Department of Fisheries and Aquaculture (DEPAq) of the Federal Rural University of Pernambuco (UFRPE), Recife, Brazil. All procedures were performed in accordance with the Animal Ethics Committee at the Federal Rural University of Pernambuco (permit number 058/2019).

Five mature females (total length 9.7 cm ± 0.6, weight 16.6g ± 3.2) and five males (total length 7.2 cm ± 0.5, weight 4.8g ± 1.2) were selected and induced by intraperitoneal injection (just below the pectoral fin) of Ovopel® [(D-Ala6, Pro9-Net) - mGnRH + metoclopramide] (5 mg/kg for males and females). After hormone application, the breeders were transferred to 40-liter tanks in a ratio of 1 male: 1 female (one couple per tank). After spawning, the broodstock were removed and the eggs were kept with constant aeration and water renewal. Water quality parameters remained within acceptable values for the species during spawning and embryogenesis, mean temperature was maintained around 28 ºC ± 0.05 and pH at 7.1 ± 0.09.

On the second day post-hatching (dph), the larvae from the five couples were mixed in one tank and were randomly selected to avoid any family effect. Four treatments were tested with four replicates each, for a total of 16 tanks. One hundred larvae were distributed in each of the 16 polyethylene tanks of 40 L at a stocking density of 2.5 larvae/L. Each tank had an individual biological filter with a filtration capacity of 86 liters/h and constant aeration.

At three dph, *ad libitum* feeding was initiated with a diet containing 55% crude protein (CP) supplemented with different doses of MT (0, 20, 40, and 60 mg 17α-methyltestosterone/kg feed) for 30 days, the same period tested before for direct hormonal sex reversal with estradiol-17β (Bem et al., 2012). Fish were fed five times a day. In addition, during the first 20 days (3 to 22 dph), plankton from fertilized pond water filtered in an 80 µm mesh and freshly hatched brine shrimp nauplii (*Artemia franciscana*) were offered at a density of 500 nauplii/larvae/day, divided into three feedings. During the experiment, dissolved oxygen, temperature, and pH values were measured daily, and ammonia and nitrite values were measured weekly. Partial water changes were made when necessary.

After the 30-day period of hormone treatment, the individuals were fed twice daily exclusively with feed containing 45% CP without the addition of hormones and kept in the tanks for another 90 days.

At 122 dph, when the individuals were already sexually mature, 30 fish from each treatment were euthanized by immersion bath with eugenol to evaluate the efficiency of the hormone treatments, and final body weight, body depth (from the dorsal margin of the body to the ventral margin of the body, at the base of the pelvic fin where it attaches to the body), and total length were measured. Survival was evaluated at 32 and 122 dph.

### Preparation of the feed supplemented with MT

Three diets were prepared with different doses of the hormone 17α-methyltestosterone (20, 40, and 60 mg/kg feed) dissolved in 95% ethanol using the ethanol evaporation method (Celik et al., 2011). In addition, glycerin (0.5%/kg by volume) was added in order to prevent any harmful effects of the alcohol (El-Greisy and El-Gamal, 2012). After drying, the feed was divided into portions, packed in plastic bags and refrigerated, removing portions for immediate consumption. In the control treatment, 95% ethanol and glycerin were added to the feed, following the same procedure for drying and storage.

### Sexing and histology

Gonads were evaluated at 122 dph for 30 individuals of each treatment for sex identification. The individuals were firstly classified based on gonads macroscopic morphology, as females when the presence of oocytes occupying a large part of the abdominal cavity was observed, while the males had whitish, opaque, and thin gonads (Nascimento et al., 2017). Individuals that exhibited both male and female gonads simultaneously were classified as intersex, and those that had undifferentiated gonads were classified as indeterminate.

Afterwards, those 30 individuals had their gonads evaluated histologically. For this, the samples were fixed in 2.5% glutaraldehyde and 2.5% paraformaldehyde in 0.1 M phosphate buffer (pH 7.4). The gonads were dehydrated in a graded series of ethanol, followed by immersion in 1-2-propylene oxide, and finally embedded in resin. Sections of 4 µm thick were made with a glass knife, which were stained with toluidine blue and examined under a light microscope.

### Micronuclei and erythrocyte nuclear alterations

To evaluate a possible genotoxic effect caused by 17α-methyltestosterone, 20 individuals of each treatment at 122 dph had peripheral blood collected via tail vein puncture with a 1.0 ml heparinized syringe. Blood smears were immediately prepared with one drop of blood (~50 µl). The slides were dried at room temperature and then stained by the Panoptic fast staining technique (Laborclin). For each individual, two smears were prepared, and 1,000 erythrocytes were examined on each with 1,000 x magnification to determine the frequency of micronuclei and other nuclear alterations.

The evaluation of the frequency of micronuclei and other nuclear alterations were classified as described by Carrasco et al. (1990) for fish erythrocytes; micronucleus: is round or ovoid and has about one fifth of the size of the main nucleus, are non-refractory particles and present the same color and staining intensity as the main nucleus; binucleated: presents two nuclei in the same cell; blebbed nucleus: the nucleus shows a small evagination of the nuclear envelope; lobed nucleus: the nucleus presents larger evaginations than the blebbed; and notched nucleus: the nucleus presents a notable cut in the content of the nuclear material. Another type of nuclear alteration, which presents a constriction similar to the shape of the number eight, was also observed and classified as an “eight-shaped” nucleus according to Furnus et al. (2014).

### Statistical analysis

Data for water quality, final body weight, body depth, and total length were presented as mean ± standard deviation. For all data obtained, statistical tests were applied to verify the normal distribution of data and homogeneity of variances through the Shapiro-Wilk and Levene tests, respectively. Parametric variables (total length and survival) were submitted to one-way analysis of variance (ANOVA), followed by Duncan’s test when significant differences between treatments were observed. For the non-parametric variables (body depth and body weight) the Kruskal-Wallis test was applied. The Chi-square (χ2) test was used to compare the sex ratios of each treatment with the control.

The frequencies of micronuclei and other nuclear alterations were expressed per 1,000 cells (^0^/_00_). Kruskal-Wallis was utilized to compare the frequency of nuclear alterations between the treatments and the control, followed by Mann-Whitney test with *P* value adjusted by Bonferroni when significant differences between treatments were observed. For statistical analysis, micronuclei, which are standard indicators of genotoxicity, were considered separately from other nuclear alterations, and the nuclear alterations were considered together. Statistical analyses were performed using R v. 4.0.2 (R Core Team, 2020) and a 5% significance level was adopted for all tests.

## Results

Throughout the experiment, the mean values of temperature (28.03 ºC ± 0.05), pH (7.14 ± 0.09), dissolved oxygen (6.68 mg/l ± 0.04), ammonia (0.67 ± 0.09), and nitrite (0.82 ± 0.32) remained within acceptable values for the species in all treatments.

At 122 dph, the values of total length, body depth and body weight did not show significant differences among treatments (*P* >0.05), with means of 5.97 cm (± 0.06), 1.53 cm (± 0.03), and 2.65 g (± 0.13), respectively. Survival ranged from 62% to 75% at 32 dph and 49% to 62% at 122 dph, differing between treatments at both times (*P* <0.05) (Table 1).

**Table 1:** Growth performance and survival of yellowtail tetra (*Astyanax lacustris*) subjected to different concentrations of masculinizing hormone 17α-methyltestosterone (MT) supplemented in the feed. Data are represented as mean ± standard deviation of total length, body depth and body weight at 122 dph.

	**Control**	**20 mg/kg**	**40 mg/kg**	**60 mg/kg**
Total length (cm)	6.06 ± 0.65	5.92 ± 0.53	6.01 ± 0.70	5.89 ± 0.53
Body depth (cm)	1.75 ± 0.62	1.51 ± 0.23	1.56 ± 0.25	1.51 ± 0.15
Body weight (g)	3.15 ± 1.27	2.61 ± 0.72	2.80 ± 0.97	2.54 ± 0.62
Survival (32 dph)	62%^a^	65%^ab^	68%^b^	75%^c^
Survival (122 dph)	49%^a^	51%^ab^	54%^b^	62%^c^

dph: days post-hatching. Different letters in the same row indicate significant differences observed using Duncan’s test (p<0.05).

**Figure 1:**
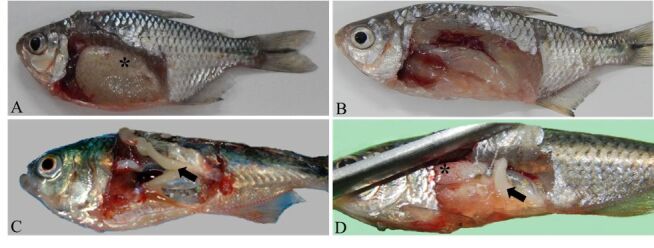
Representation of sexual classification based on primary characters for (A) female (*- ovary), (B) indeterminate, (C) male (arrow- testis) and (D) intersex (*- ovary and arrow- testis) of yellowtail tetras (*Astyanax lacustris*) treated with different concentrations of the masculinizing hormone 17α-methyltestosterone supplemented in the feed.

**Figure 2:**
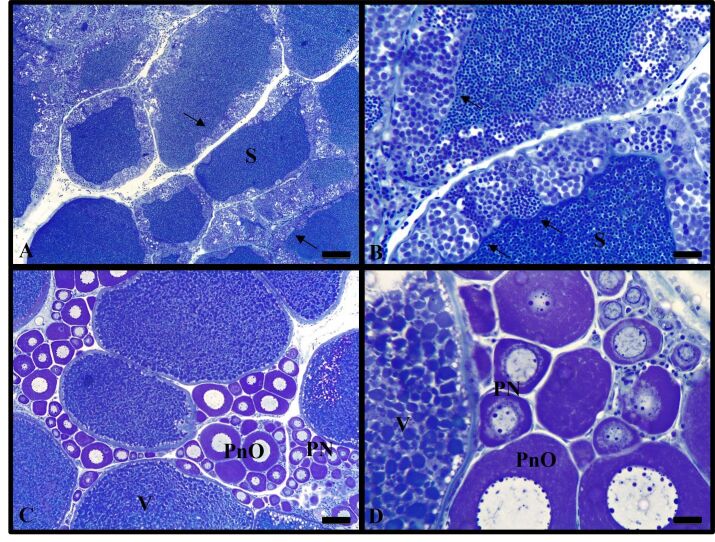
Testicular and ovarian histological section of yellowtail tetra (*Astyanax lacustris*). (A, B) male gonad (S: sperm; arrow: cysts in different stages of spermatogenesis); (C, D) female gonad region with follicles in different stages of development (PN: perinuclear follicle; PnO: previtellogenic oocytes with perinuclear nucleolus; V- vitellogenic follicle). Scale bars: 100 µm.

For the 30 individuals from each treatment that had their gonads evaluated, it was possible to identify males, females, intersex, and indeterminate individuals (Figure 1). In the control treatment, 46.7% of males and 53.3% of females were observed, while in the MT treatments the percentage of males ranged from 50% (40 mg/kg) to 76.7% (20 mg/kg) and females varied from 3.3% (20 mg/kg and 60 mg/kg) to 46.7% (40 mg/kg) (Table 2). Individuals with intersex gonads were observed in the 20 and 60 mg/kg treatments and individuals with undifferentiated gonads were observed in all MT treatments. Gonads at different stages of development (from immature through spawning capable, Araújo et al., 2019) were observed in all treatments (Figure 2). There was a significant difference (*P* <0.05) between the frequencies of males and females found in the 20 and 60 mg/kg MT treatments and in the control (Table 2).

**Table 2:** Sex ratio found in treatments without hormone (control) and with the addition of different dosages of 17α-methyltestosterone in *Astyanax lacustris* at 122 days post-hatching.

**MT dose (mg/kg)**	**Male**	**Female**	**Intersex**	**Indeterminate**	**χ2**
0 (control)	14 (46.7%)	16 (53.3%)	0	0	
20 mg/kg	23 (76.7%)	1 (3.3%)	1 (3.3%)	5 (16.7%)	* p=0.0001
40 mg/kg	15 (50.0%)	14 (46.7%)	0	1 (3.3%)	NS p=0.6976
60 mg/kg	17 (56.7%)	1 (3.3%)	4 (13.3%)	8 (26.7%)	* p=0.0008

* Significant difference between treatment and control (p<0.05). NS: non-significant.

**Figure 3:**
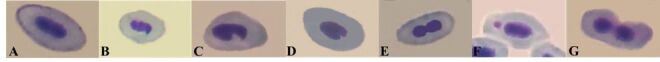
Micronuclei (MN) and erythrocyte nuclear alterations (NA) identified in yellowtail tetra (*Astyanax lacustris*) treated with different concentrations of 17α-methyltestosterone supplemented in the feed. Erythrocyte with normal nucleus (A), lobed nucleus (B), notched nucleus (C), blebbed nucleus (D), eight-shaped nucleus (E), micronucleus (F), and binucleated (G). 1000x magnification.

Regarding micronuclei and erythrocyte nuclear alterations, it was possible to observe all six types of alterations in all treatments and in the control, except for binucleated erythrocyte, which was not observed in the control treatment (Figure 3). Although the mean MN values showed a tendency to increase directly proportional to the MT dose, no significant differences were found between the treatments and the control (Table 3). The 40 mg/kg and 60 mg/kg treatments obtained the highest NA values, differing significantly from the control.

**Table 3:** Mean ± standard deviation of micronuclei (MN) and nuclear alterations (NA) in *Astyanax lacustris* treated with different dosages of 17α-methyltestosterone at 122 days post-hatching.

**MT dose (mg/kg)**	**MN**	**NA**
0 (control)	0.40 ± 0.52	3.46 ± 1.58^a^
20	0.70 ± 0.48	5.44 ± 1.08^a^
40	1.30 ± 1.16	5.66 ± 1.34^b^
60	1.30 ± 0.95	6.34 ± 1.68^b^

Mean value for 1000 erythrocytes. Means followed by different letters in the same column indicate significant differences using Mann-Whitney test with *p* value adjusted by Bonferroni.

## Discussion

The use of MT had a positive influence in obtaining males of *A. lacustris* without affecting survival. A sex ratio of more than 50% males was obtained at all doses of MT used, with the lowest dose (20 mg/kg) being the most successful one, reaching more than 75% of males. Contrastingly, the highest dose (60 mg/kg) resulted in a higher number of indeterminate and intersexed individuals, which would have been expected if the dosage were too low. As no fish with undifferentiated gonads were found in the control treatment, this delay in maturation or sterility of the individuals may have been caused by the MT.

Other studies with masculinization using MT supplemented feed in different fish species, obtained a higher percentage of males as in the case of tilapia where it was possible to obtain 94% of males with 60 mg/kg (Silva et al., 2022) and for silver perch where 100% of males were obtained with 9 mg/kg (Sulaeman and Fotedar, 2017). In addition, a male percentage similar to ours was described for *Rhamdia quelen* with 72% males at a dose of 60 mg/kg (Weiss et al., 2018a). There is not always a linear relationship between increasing the MT dosage and improving the masculinization rate. In fact, treatments with excessive doses, which in our study seems to be the case of treatments 40 and 60 mg/kg, can lead to disruptions in gonadal development or sterility (Devlin and Nagahama, 2002) and could also lead to reduced masculinization, or in some instances, it could induce paradoxical feminization (Demska-Zakes and Zakes, 1997; Marjani et al., 2009; Amer et al., 2021, Karaket et al., 2023).

In some species, when hormone-induced sex reversal was performed in the early stages, feminization with estrogens showed better results than masculinization with androgens (Bombardelli and Hayashi, 2005). When direct feminization was employed at an equivalent exposure period to that of this study and under the same form of supply, 76% of females were obtained at a dose of 40 mg/kg estradiol-17β (Bem et al., 2012). The failure to obtain full reversal (100%) in both cases may be due to the dosage and duration of hormone treatment. Moreover, according to Pandian and Sheela (1995) and Weiss et al. (2018a), the use of high hormonal doses could cause sterility which may explain the presence of adult individuals with undifferentiated gonads in our study.

The presence of intersex individuals in the MT treatments may also be an indication that the time of hormone administration was not sufficient to cover the labile period (Weiss et al., 2018b) (i.e., the sensitive period in the early life stages during which the sexually undifferentiated gonad differentiates into male (testicular tissue) or female (ovarian tissue) gonad (Budd et al., 2015)), for masculinization in *A. lacustris*. As previously mentioned, this period has not yet been determined for *A. lacustris*, but for the sister species, *A. altiparanae*, the differentiation into ovaries and testes occurs at 58 and 73 dph, respectively (Adolfi et al., 2015). Thus, to obtain a higher rate of masculinization, the optimal dose found in this study (20 mg/kg) must be used for a period longer than 30 days to cover the entire labile period.

The presence and increased frequency of micronuclei in erythrocytes is an indicator of genetic damage, likewise, nuclear alterations in erythrocytes may be an indicator of cytotoxic effect, both caused in fish exposed to environmental and chemical contaminants (Samanta and Dey, 2012; Ribeiro et al., 2014; Viana et al., 2018). Regarding the toxicity of MT in *Astyanax*, other studies observed an increased frequency of NA when adult individuals of another sister species, *A. bimaculatus*, were treated with feed supplemented with 60 mg/kg of this hormone for 28 days (Rivero-Wendt et al., 2013a) or by immersion with 1 mg for 96 hours (Rivero-Wendt et al., 2013b). In our study, three months after the end of the 30-day period of exposure to MT, only the highest doses of MT (40 and 60 mg MT/kg) showed a significant increase in NA frequency. The treatment of 20 mg MT/kg, which presented the highest percentage of males, was not significantly different from the control treatment regarding to MN and NA, suggesting that MT use at low doses and long-term exposure during the early stages of development does not cause a genotoxic effect for *A. lacustris*.

In relation to the neomales candidates’ fertility, even though Rivero-Wendt et al. (2013a) observed a reduction in spermatozoa production in the sister species, *A. bimaculatus,* treated with MT, it may be due to the fact that the individuals used in their study were adults, with the gonads already differentiated, unlike our one in which individuals were treated with MT at early stages of development, before sex differentiation. In addition, in our study, MT does not seem to affect the development of the individuals and probably it is not the cause of not achieving 100% sex reversion. Gonads from neomales candidates did not present malformation and look similar to the nontreated (control) individuals, which may indicate that possibly it will not affect their fertility.

Thus, the use of feed enriched with 17α-methyltestosterone at a dose of 20 mg/kg during the first 30 days of feeding of yellowtail tetra (*A. lacustris*) shows the possibility to be beneficial for achieving neomales.

## Conclusion

In order to complement the results achieved, further studies would be interesting to evaluate whether the time of exposure to MT and if lower doses of it influence the rate of masculinization. It remains necessary to conduct progeny tests to identify the neomales individuals that can be used in an all-female production free of hormones. The results of this study indicate that it is possible to achieve a male percentage of 76.7% in yellowtail tetra (*A. lacustris*) by feeding feed enriched with 17α-methyltestosterone at a dose of 20 mg/kg during the first 30 days of feeding.
